# VATS Pneumonectomy: The Posterior Approach

**DOI:** 10.1186/1749-8090-10-S1-A350

**Published:** 2015-12-16

**Authors:** D Miller, M Will, W Walker

**Affiliations:** 1 Department of Cardiothoracic Surgery, Royal Infirmary of Edinburgh, Edinburgh, UK

## Background/Introduction

VATS lobectomy is a well-established procedure with proven benefit for patients, in the form of fewer complications and shorter hospital stays but providing equivalent clearance of tumours compared to thoracotomy. A less common and more complex procedure is performing pneumonectomy by the same technique, with little published evidence to demonstrate the same clinical or oncological benefits.

## Aims/Objectives

Our objective was to review the characteristics and outcomes for patients undergoing VATS pneumonectomy at a single institution.

## Method

Pneumonectomy was performed by the previously described posterior Edinburgh VATS approach. This technique is optimised with the use of a 30 degree HD camera, and dividing hilar structures in the following order: SPV, main PA, IPV, and main bronchus after sampling of station 7 lymph nodes. Operation notes, discharge summaries and pathology reports of patients who have undergone VATS pneumonectomy from 1992 until the present were reviewed. The details of these patients were recorded in a worksheet and analysed using Microsoft Excel.

## Results

Age (mean, range) 63 (14-82) Female 9 (45%) Right Pneumonectomy 5 (25%) Completion Pneumonectomy 3 (15%) Operation Time (Mean ± SEM, mins) 203 ± 47 Blood Loss (Mean ± SEM, mls) 205 ± 174 Length of Stay (Median, range, days) 6 (3-19) Major Complications 1 (5%) Minor Complications 5 (25%) 30 day Mortality 0 (0%) Pathology (NSCLC) 18 (90%) TNM 7th Ed Stage IIIa or less 19 (95%) Complete Resection (R0) 19 (95%)

## Discussion/Conclusion

VATS pneumonectomy is a safe procedure when performed by an experienced VATS surgeon. Patients with large central tumours, tumours crossing the fissure or endobronchial lesions not amenable to sleeve resection are suitable candidates for this procedure. This complex procedure achieves excellent oncological clearance and appears to have a low complication rate.

